# European Hospitals’ Transition Toward Fully Electronic-Based Systems: Do Information Technology Security and Privacy Practices Follow?

**DOI:** 10.2196/11211

**Published:** 2019-03-25

**Authors:** Sylvestre Uwizeyemungu, Placide Poba-Nzaou, Michael Cantinotti

**Affiliations:** 1 Accounting Department Université du Québec à Trois-Rivières Trois-Rivières, QC Canada; 2 Department of Organization and Human Resources Management École des Sciences de la Gestion Université du Québec à Montréal Montréal, QC Canada; 3 Psychology Department Université du Québec à Trois-Rivières Trois-Rivières, QC Canada

**Keywords:** health information technology, data security, patient data privacy, health services, electronic health records

## Abstract

**Background:**

Traditionally, health information has been mainly kept in paper-based records. This has deeply changed throughout approximately the last three decades with the widespread use of multiple health information technologies. The digitization of health care systems contributes to improving health care delivery. However, it also exposes health records to security and privacy breaches inherently related to information technology (IT). Thus, health care organizations willing to leverage IT for improved health care delivery need to put in place IT security and privacy measures consistent with their use of IT resources.

**Objective:**

In this study, 2 main objectives are pursued: (1) to assess the state of the implementation of IT security and privacy practices in European hospitals and (2) to assess to what extent these hospitals enhance their IT security and privacy practices as they move from paper-based systems toward fully electronic-based systems.

**Methods:**

Drawing on data from the European Commission electronic health survey, we performed a cluster analysis based on IT security and privacy practices implemented in 1723 European hospitals. We also developed an IT security index, a compounded measure of implemented IT security and privacy practices, and compared it with the hospitals’ level in their transition from a paper-based system toward a fully electronic-based system.

**Results:**

A total of 3 clearly distinct patterns of health IT–related security and privacy practices were unveiled. These patterns, as well as the IT security index, indicate that most of the sampled hospitals (70.2%) failed to implement basic security and privacy measures consistent with their digitization level.

**Conclusions:**

Even though, on average, the most electronically advanced hospitals display a higher IT security index than hospitals where the paper system still dominates, surprisingly, it appears that the enhancement of IT security and privacy practices as the health information digitization advances in European hospitals is neither systematic nor strong enough regarding the IT-security requirements. This study will contribute to raising awareness among hospitals’ managers as to the importance of enhancing their IT security and privacy measures so that they can keep up with the security threats inherently related to the digitization of health care organizations.

## Introduction

### Motivation and Objectives

In many countries, health care services delivery is being reformed—and some say revolutionized [1]—through information technology (IT). IT potential is being leveraged in the quest to achieve what has been called the *triple aim* [2-4], that is, (1) improving individual care experience, (2) improving population health, and (3) reducing per capita cost of health care.

IT developments have led to the digitization of health records, thus offering new or improved means to efficiently and effectively collect, process, store, consult, and share health information. Digitized, health information becomes more portable and readily shareable within and among different health care organizations; it becomes readily available to public health administrators for health surveillance and policy-making purposes; it becomes, under certain conditions, available for research; it also becomes more accessible to patients. Thus far, the significant majority of the literature suggests positive effects of digitization on the effectiveness of health care outcomes [5]. However, despite all those advantages, the digitization of health information exposes health records to security breaches inherently related to IT [6]. Indeed, potential users of health IT (HIT) express various concerns over IT-related security and privacy issues [7], and these concerns may negatively affect the trust of potential HIT users. As a result of this decrease in trust, patients, as well as health care professionals, may be reluctant to widely use some HIT functionalities, such as health information exchange (HIE), telehealth, and mobile health [8], threatening the necessary “meaningful use” of HIT [9]. The ultimate result would be ineffective health care delivery [10,11], as well as ineffective public health monitoring [12] or health research [13,14].

To alleviate security and privacy concerns, health care organizations willing to leverage IT for improved health care delivery need to put in place IT security measures consistent with their IT development plans. In this study, our objective was twofold: to assess the state of the implementation of IT security and privacy practices in European hospitals and to assess to what extent European hospitals enhance their IT security practices as they move from paper-based systems toward fully electronic-based systems.

### Background and Significance

Traditionally, health information has been mainly kept in paper-based records. This has deeply changed throughout approximately the last three decades, with the widespread use of multiple HIT, an umbrella term we use here to refer to all IT systems used for storing, accessing, processing, sharing, transmitting health information, or for supporting health care delivery and health care systems management. Thus defined, HIT encompasses all the 4 functionality-based categories of IT proposed by Adler-Milstein et al [15]: provider-centric electronic record, patient-centric electronic record, HIE, and telehealth.

By their mere nature, HIT compiles a wide range of highly sensitive information. This information includes not only current data related to patients’ tests, diagnoses, and treatments but also past medical history [16]. Health care providers need to keep and manipulate that information securely not only to meet the patients’ willingness to keep their health information private but also to live up to the health care organizations’ moral and legal responsibilities. However, the task of keeping health records secure is affected by the dynamic nature of the HIT environment.

In recent years, the landscape of security of health records has changed following a number of phenomena related to the digitization. For example, professional and academic literature has extensively echoed security issues arising from IT trends such as hosting health records on distant servers operated by third-party cloud services providers [17,18], the usage of mobile devices, and the related trends of bring your own device in health care [19-21], as well as mobile health apps [22,23] and the IT-enabled HIE [24,25].

### Information Technology Security Incidents in Health Care Settings

A report by Infosec Institute underscored that the remarkable growth in the adoption of electronic health records (EHRs) in the last years has not been accompanied by a parallel evolution in cybersecurity measures, thus rendering the health care industry ill-equipped and poorly protected with regard to cyber threats [26]. This bleak assessment seems to be upheld by numerous reports of IT-related incidents in hospital settings. According to a 2014 survey by Information Security Media Group (ISMG), at least one security breach that affects fewer than 500 individuals has occurred in 75% of surveyed health care organizations in the United States, and at least one incident affecting more than 500 individuals has been reported by 21% of surveyed health care providers [27]. In the 2015 survey by Healthcare Information and Management Systems Society, two-thirds (68%) of surveyed health care organizations in the United States reported having recently experienced a significant security incident [28]. Reported security incidents came both from external threats (63.6% of health care organizations) and insider threats (53.7%) [28].

These statistics of IT-related security breaches in health care settings are disturbing, and the reality may be even bleaker when one considers that many security incidents remain undetected or are not properly assessed [27], as well as the propensity of organizations to underreport security incidents [29].

Documented incidents show that security breaches in health care settings can be expensive. For example, Absolute Software Corporation reported cases of breaches in health care data that cost hospitals from US $250,000 to US $2.5 million in settlement payments. Even though these amounts are quite sizeable, they represent but a fraction of the overall financial burden of the incidents [30].

Security and privacy concerns, as well as the fear of related liabilities, may prevent health care providers from leveraging IT for improving their services. Increasing HIT security and privacy practices in hospitals is then an important step forward for effective health care delivery.

### Health Information Technology–Related Security System

In response to IT-related security and privacy concerns, health care providers who adopt HIT need to put in place an adequate security system. This system is “a set of security mechanisms that are implemented according to a security policy,” which is “a collection of rules that allow or disallow possible actions, events, or something related to security” [10].

Generally speaking, an IT security policy aims at ensuring that an organization’s IT assets (hardware, software, data, and people) respond constantly to required levels of confidentiality, integrity, and availability [31,32]. These 3 basic IT security requirements are generally referred to as the CIA triad (Confidentiality, Integrity, Availability) [33].

The notion of confidentiality is generally defined as “restricting information to persons belonging to a set of specifically authorized recipients [34].” Confidentiality requires that only duly authorized people can get access to data, whether they are stored, being transmitted, or being treated. This can be achieved through encryption of data or through controlled access to the systems. These are technological means, but confidentiality can also be achieved relying on moral dispositions (eg, professional silence) [34]. With regard to encryption, the 2014 survey of ISMG showed that although encryption is commonly applied for health data transmitted across exposed networks, it is less applied to data stored in mobile devices and other storage media [27]. The confidentiality requirement responds to privacy concerns that are of paramount importance in health care systems given the sensitivity of information they contain.

With the integrity criterion, it is expected “that information is protected against unauthorized modification or deletion as well as irrevocable, accidental, and undesired changes by authorized users” [33].

As for availability, it requires that a system be accessible and fully operational whenever an authorized user needs to utilize it. The availability criterion refers to multiple aspects ranging from scalability (adaptability to changing performance needs) to resilience (resistance to software or hardware failures) and to recoverability of data in case of loss for whatever reason [33].

## Methods

### Data Source

We used data from the European Commission 2013 electronic health (eHealth) survey (Joint Research Centre, Institute for Prospective Technological Studies). The objective of the survey was “to benchmark the level of eHealth use in acute care hospitals in all 27 European Union member states, Croatia, Iceland, and Norway” [35]. The dataset used might seem outdated but we deem it still relevant. First, the survey we are referring to is the last one of this magnitude to have been conducted at the level of the European Union. It is thus the most recent. Second, it has been demonstrated that secondary data that are 5 years old or even older can provide meaningful, empirically grounded, and useful insights in the field of IT security in the health care sector [36]. Third, over the last 5 years, the HIT security field has not recorded significant changes that would render our data and our analysis obsolete. The analysis of recent literature reviews of IT security in health care settings [37-39] shows that the health care industry still lags behind in IT security measures implementation. It also shows that there is no major technical breakthrough in HIT security and no notable new-brand threats. One can only note a greater awareness among health care professionals and patients following multiple breaches of health data made public and the strengthening of legal requirements. In this regard, the adoption of the General Data Protection Regulation throughout the European Union territory has been too recent for its effects on IT security and privacy practices in the health sector to have been felt. This regulation came into force in May 2018 [40]. Fourth, other factors specific to the hospital context suggest a slow pace of changes in such a context. Many small health care organizations lack financial and human resources for undertaking substantive (as opposed to symbolic) IT security programs [41]. For their part, large health care organizations tend to be complex systems in their structure and management [42,43], and in such systems, cultural shifts to implement security and privacy measures may take a lot of time.

### Sample

To ensure the representativeness of the sample, the survey team undertook the following steps [44]. They estimated the universe of acute care hospitals in the European Union, combining various sources (previous survey, lists of hospitals from the World Health Organization, and national ministry of health of each covered country). The estimation yielded a universe of 8199 acute care hospitals. From this point, the survey team proceeded to a stratification sampling procedure to ensure geographical representation (Nordic countries, Southern Europe, Western Europe, and Central and Eastern Europe). The stratification process also included other considerations related to the hospital’s ownership (public, private, and other) and size (number of beds). To guarantee the representativeness of the sample at the end of data collection, the survey team proceeded to nonresponse rate corrections. In total, the survey team contacted 5424 acute care hospitals, and 1753 hospitals completed the interview [45]. This corresponds to a rate response of 32.3%.

Of the 1753 initial observations, only 30 (1.7%) were dropped because of missing data (“don’t know” response or no answer at all) on key variables, which led us to a final sample of 1723 European hospitals. Even though the portion of dropped cases is very low, we performed statistical analyses to assess whether the dropped observations were significantly different in any way from the retained observations. Little’s missing completely at random test (χ^2^_5,1723_=7.8, *P*=.17) indicated that ignoring cases with missing values does not a priori bring about a systematic bias. However, we went a step further and performed a nonresponse bias analysis comparing key characteristics of dropped cases against sampled cases (Table 1). To do so, we used Fisher exact test of homogeneity instead of chi-square test, thus taking into account the fact that some cells of the contingency table would contain low expected frequencies (<5), especially for the group of dropped cases (n=30). The results showed that the dropped cases are not significantly different from the sampled cases with regard to all of hospital characteristics.

Table 1 depicts descriptive statistics of our sample. Public hospitals account for 70.38% (1188/1688) of surveyed hospitals. Most hospitals in our sample are not university hospitals (1485/1723; 86.19%). Independent hospitals operating either on 1 site (710/1723; 41.21%) or on multiple sites (542/1723; 31.46%) make up 72.67% (1252/1723) of the sample. More than half (857/1568; 54.66%) of surveyed hospitals can be qualified as small (250 or fewer beds), whereas a small portion (167/1568; 10.65%) falls in the category of large hospitals (more than 750 beds). With regard to IT budget, a large majority (1086/1260; 86.19%) of sampled hospitals allocate 3% or less of their total budget to their IT function. Hospitals that devote more than 5% of their total budget to IT represent a negligible portion of the sample (52/1260; 4.13%). Almost 7 out of 10 hospitals (1159/1665; 69.61%) have in place an in-house, designed IT security regulation; 6 out of 10 (997/1665; 59.88%) report relying on a nationally-designed regulation, whereas a regional regulation is referred to by less than 3 out of 10 hospitals (475/1665; 28.53%).

**Table 1:** Characteristics of sampled versus nonsampled hospitals.

Variable and characteristics		% of nonsampled (n=variable), n (%)	% of sampled (n=variable), n (%)	Chi-square (*df*)	*P* value
**Status**		**N=28**	**N=1688**	0.2 (2)	.91
	Public	20 (71)	1188 (70.38)		
	Private	5 (18)	335 (19.85)		
	Not for Profit	3 (11)	165 (9.77)		
**University hospital**		**N=30**	**N=1723**	1.3 (1)	.42
	Yes	2 (7)	238 (13.81)		
	No	28 (93)	1485 (86.19)		
**Single/multiple sites**		**N=30**	**N=1723**	1.7 (4)	.77
	Independent/One site	14 (47)	710 (41.21)		
	Independent/Multiple sites	9 (30)	542 (31.46)		
	Part of a group of hospitals	4 (13)	341 (19.79)		
	Part of a group of care institutions	2 (7)	78 (4.53)		
	Other	1 (3)	52 (3.02)		
**Size (number of beds)**		**N=25**	**N=1568**	7.4 (3)	.05
	<101	11 (44)	363 (23.15)		
	101 to 250	8 (32)	494 (31.51)		
	251 to 750	6 (24)	544 (34.69)		
	>750	0 (0)	167 (10.65)		
**IT^a^** **budget (% of total hospital budget)**		**N=19**	**N=1260**	1.6 (3)	.62
	<1%	10 (53)	486 (38.57)		
	1% to 3%	7 (37)	600 (47.62)		
	3.1% to 5%	2 (11)	122 (9.68)		
	>5%	0 (0)	52 (4.13)		
**Security regulation**		**N=23**	**N=1665**		
	National	11 (48)	997 (59.88)	1.4 (1)	.29
	Regional	3 (13)	475 (28.53)	2.7 (1)	.16
	Hospital	13 (57)	1159 (69.61)	1.8 (1)	.18

^a^IT: information technology.

**Table 2:** Security and privacy practices measures.

Variable		Measure (Yes or no)
**Confidentiality: which of the following security measures are taken to protect the patient data stored and transmitted by the hospital’s IT^a^** **system?**		
	Stored data	Encryption of stored data
	Transmitted data	Encryption of transmitted data
	Access control	Workstations with access only through health professional cards or codes
Integrity		Is data entry in the hospital’s IT system certified with digital signature?
Availability		Is your IT team able to immediately restore critical clinical information system operations if a disaster causes the complete loss of data at your hospital’s primary data center?

^a^IT: information technology.

### Measurement

For contextual variables, measures used are depicted in Table 1, column “characteristics.” The level attained by a hospital in the transition from a paper-based system to a fully electronic-based system was measured on a 9-point Likert scale (1=totally paper-based and 9=totally electronic-based, with point 5 as a hybrid model). For simplicity, this measure has been transformed into 3 nominal categories: hospitals that chose positions from 1 to 3 were qualified as having a paper-dominant system; a hybrid system label was given to hospitals that chose positions from 4 to 6, and the remaining hospitals (positions from 7 to 9) were deemed to have an electronic-dominant system. We present in Table 2 the measures used to capture the implementation of HIT security and privacy practices used as our clustering variables. These practices were measured through a dichotomous scale (eg, coding “1” if a confidentiality-related practice is implemented and “0” when it is not implemented).

To check for possible variables multicollinearity before performing our cluster analysis [46], we produced a correlation matrix of all our variables (clustering variables and contextual variables; Multimedia Appendix 1). The results show that the risk of multicollinearity is very low: all the correlations coefficients are very low, the maximum correlation coefficient noted being .359 (between encryption of stored data and encryption of transmitted data). Besides, multicollinearity diagnostics (Multimedia Appendix 1) show that the tolerance values vary between .78 and .96, far above the commonly used cutoff threshold of .1 [47]. The highest variance inflation factor noted is equal to 1.28, far below the usual cutoff threshold of 10.0 [47]. These values show that multicollinearity is not a concern in our data.

To assess the state of the implementation of IT security and privacy practices in European hospitals, we developed an IT security index for each hospital. We developed the IT security based on the responses given by hospitals with regard to their security practices. We considered 5 questions asked (see Table 2). A given hospital had to respond whether or not it has implemented the security practice related to confidentiality (3 questions), to integrity (1 question), and to availability (1 question). With a “Yes” response, a hospital scored 1 point and 0 otherwise (a “No” answer). The IT security index reflects the total score obtained by each hospital for the 5 questions. However, considering that the confidentiality component was measured through 3 questions, whereas the other 2 components (integrity and availability) were measured each by 1 question, we estimated that the score obtained by simply summing up the results from the 5 questions would be skewed in favor of the confidentiality component. In the absence of theoretical or empirical evidence to the effect that any of the 3 components of the security triad (confidentiality-integrity-availability) would be more significant when compared with the 2 others, we hypothesized an equal weight for the 3 components. That is why the confidentiality component accounts for 1 in the index even if it is measured through 3 questions: each of the 3 questions related to confidentiality accounted for one-third (1/3) of the value of confidentiality in the index. As the integrity and availability components account each for 1, the maximum score of the IT security index for a given hospital is 3 (for a hospital that has all the 5 practices implemented: 1/3+1/3+1/3+1+1), and the minimum is 0 (for a hospital with none of the practices implemented).

### Cluster Analysis

We performed an agglomerative hierarchical clustering procedure combining Ward’s minimum variance criterion with the squared Euclidian distance. This procedure allows distributing the observations into distinct subgroups (clusters) in the way that maximizes at the same time the intrasubgroup similarity and the intersubgroups dissimilarity [48]. Each subgroup comprises hospitals more or less homogenous with regard to clustering criteria (in this case HIT security practices implemented), and each subgroup is highly distinct to other subgroups with regard to the same criteria.

To identify the optimal number of clusters, we first examined the Euclidian distances across the clusters in the dendrogram produced with the clustering procedure. We identified 2 apparently equally plausible solutions, a 3-cluster and a 4-cluster solution. To decide which of these 2 solutions would be better, we followed Ketchen and Shook’s [49] recommendation: we ascertained the robustness of both by replicating the clustering algorithm on subsamples of about 80%, 60%, and 40% of observations randomly selected using SPSS’s random selection functionality (SPSS version 24, SPSS Inc). The analysis of the dendrograms produced with all these subsamples suggested a slight advantage of the 4-cluster solution over the 3-cluster solution. However, when we performed a discriminant analysis test, we found that 2 clusters of the 4-solution clusters were too close, whereas all clusters in the 3-cluster solution were well-separated. Therefore, we chose the 3-cluster solution.

The discriminant analysis also allowed us to test the validity of the clusters. This test “runs the data back through the minimum-variance method as a discriminant function to see how accurately hospitals are classified [50].” The results of this test indicated a perfect classification accuracy (100%) for clusters 2 and 3 and a high level of classification accuracy (92.3%) for cluster 1. Overall, 98.7% of original observations were correctly classified. Moreover, based on cross-validation with analysis and holdout subsamples of respectively 60% (n=992) and 40% (n=731) of the total sample [51], for the 3-cluster solution, both hit ratios (97.7% for the analysis subsample and 98.1% for the holdout subsample) largely exceeded the threshold values of both maximum chance criteria (C_max_) and proportional chance criteria (C_Pro_). Indeed, the 2 hit ratios should be greater than Max (1.25[C_Pro_; C_max_]), which is the case as in this study C_Pro_=34% and C_max_=39.3%. Thus, the null hypothesis that the percentage correctly classified was not significantly different from what would be classified by chance alone was rejected.

## Results

### Implementation Level of Information Technology Security and Privacy Practices

For the whole sample, the mean IT security index is 1.26 (with an SD of 0.83), the median being 1.33. In Table 3, we present the detailed statistics of hospitals by IT security index level.

It can be noted that only a tiny fraction of hospitals (50/1723; 2.90%) display a perfect IT security index (3). The nonnegligible fraction of hospitals (225/1723; 13.06%) has no IT security and privacy practices implemented whatsoever (IT security index=0). From the column “cumulative %,” one can note the total percentage of hospitals that does not exceed a given level of the IT security index. For example, at a glance, one will see that 62.17% (1071/1723) of hospitals have achieved an IT security index of 1.33 or less. In absolute terms, these levels seem very low. Is it possible that the less electronically advanced hospitals would deem it unnecessary to implement extended IT security and privacy practices, thus displaying lower levels of IT security index? To test this hypothesis, we confronted the IT security index with the transition level toward a fully electronic-based system.

Before performing this comparison, it is worthwhile to note that a majority of sampled hospitals (1056/1723; 61.28%) consider currently using a hybrid system (transition levels 4, 5, and 6), as they were more or less halfway toward a fully electronic-based system. An electronic-dominant system is found in 26.29% (453/1723) of hospitals, whereas a paper-dominant system is found in only 12.42% (214/1723) of them.

**Figure 1:**
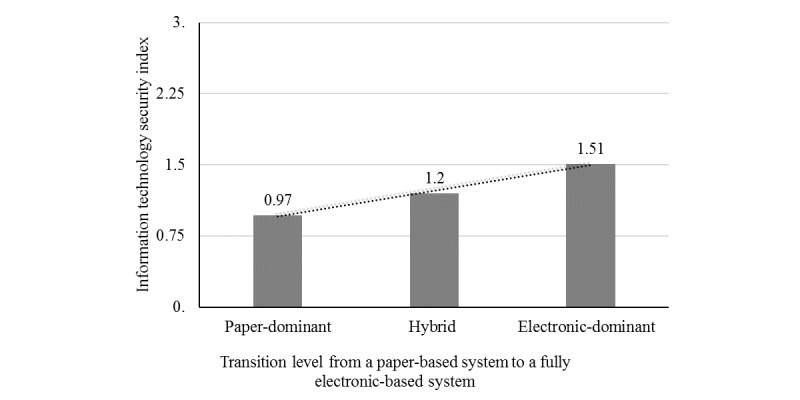
Transition level toward electronic-based system versus information technology security index-1.

In Figure 1, we present the mean scores on our IT security index (vertical axis) for different groups of hospitals according to their level in transition toward a fully electronic-based system (horizontal axis).

**Figure 2:**
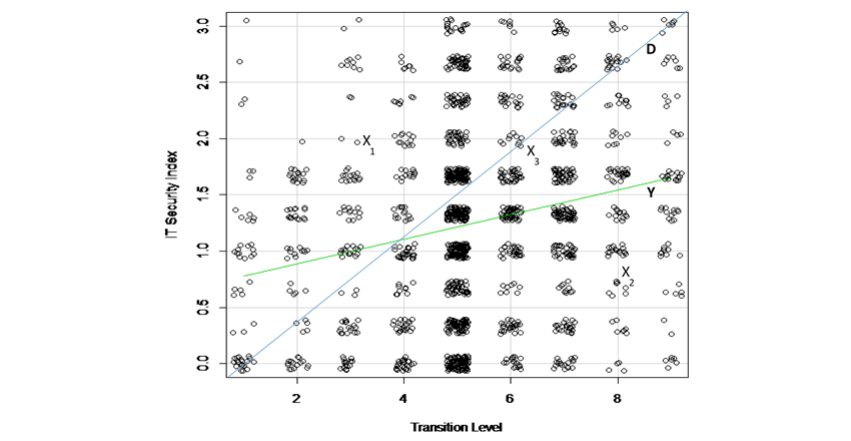
Transition level toward electronic-based system versus information technology security index-2. IT: information technology.

Globally speaking, the mean score of IT security index is low for all 3 groups constituted on the basis of their transition level to a completely electronic system. It appears that the IT security index improves as one moves from the group of hospitals with a paper-dominant system (levels 1, 2, and 3) to the group of hospitals with electronic-dominant system (levels 7, 8, and 9) through the group of hospitals in a hybrid position (levels 4, 5, and 6). However, a further analysis depicts a much more nuanced picture. For the purposes of this analysis, we produced a scatter plot (Figure 2) indicating every hospital’s coordinate (a and b) depicting its position with regard to the transition level of its system (a) and to its IT security index (b). To facilitate the reading of the coordinates, we used a “jitter” to make sure that multiple observations that fall in the same coordinate can be visualized.

**Table 3:** Information technology security index level statistics.

IT^a^ security index level	n (%)	Cumulative %
0.00	225 (13.06)	13.06
0.33	186 (10.80)	23.86
0.67	104 (6.04)	29.90
1.00	269 (15.61)	45.51
1.33	287 (16.66)	62.17
1.67	276 (16.02)	78.19
2.00	95 (5.51)	83.70
2.33	101 (5.86)	89.56
2.67	130 (7.54)	97.10
3.00	50 (2.90)	100.00
Total	1723 (100.00)	—^b^

^a^IT: information technology.

^b^Not applicable.

For facilitating the analysis of the figure, we added a diagonal line (D). If hospitals were enhancing their IT security and privacy practices as they moved forward, points representing hospitals would be scattered around the diagonal line. Instead, we found that points are scattered almost all over the surface of the figure.

Hospitals far above (far below) the diagonal line display a security index that is superior (inferior) to the average level required by their transition level toward a fully electronic-based system. For example, any hospital represented by X_1_ point has an IT-related security index above the theoretical level required by its progress toward an electronic-dominant system. Conversely, a hospital represented by X_2_ displays a security index far below the level it should attain, considering how far it has progressed toward a fully electronic-based system. The security index of hospital X_3_ is consistent with its progress in electronic-based system implementation.

The slope of the ascending curve in the figure (Y) suggests that there is a trend toward increasing IT security and privacy measures when hospitals move from a paper-based system to an electronic-based system. Though this finding is positive, it appears that the trend is not strong enough. Otherwise, the shape of the curve would be closer to the diagonal line.

### Information Technology Security Level Versus Context Variables and Paper/Electronic Transition Level

In the previous section, we tested the relationship between the level of transition toward fully electronic systems on the one hand and the level of implementation of IT security on the other. We intuitively assumed a linear relationship between the 2. In this section, we take the analysis further, not only by considering the contextual variables in addition to the paper/electronic transition level but also by testing the validity of the linear relationship assumption between the transition level and IT security level.

In Table 4 we present the results of multivariate regression analyses testing whether hospitals’ IT security levels can be predicted by hospitals’ characteristics (contextual variables) and/or their transition level from paper to electronic system.

The results from both linear models (models 1 and 2) show that hospitals’ characteristics and their transition level toward fully electronic-based system significantly contribute to the IT security level, with total R^2^ equals, respectively, to 8.6% (*F*_16,1706_=10.05; *P*=.001) and 12.0% (F_1,1705_=65.12; *P*=.001). With models 3 and 4, we tested alternative nonlinear models and concluded that they do not bring about any significant contribution.

### Results of Cluster Analysis

**Figure 3:**
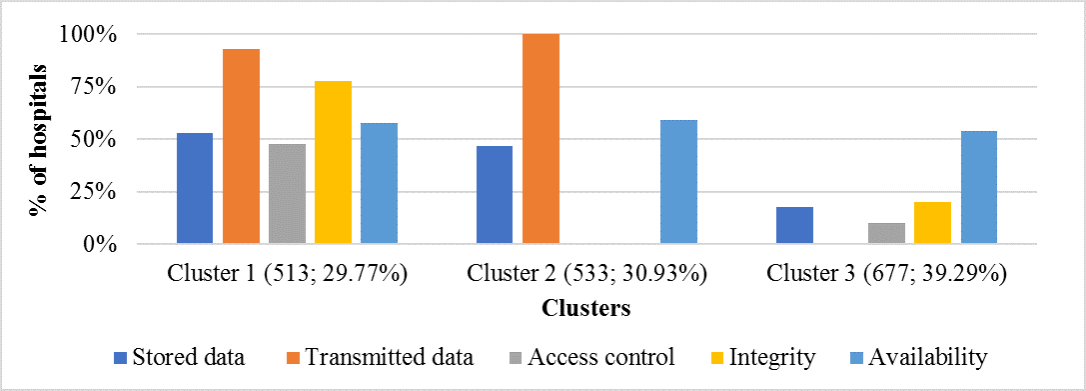
Implementation levels of information technology security practices in hospitals by cluster.

The 3 patterns of HIT-related security practices resulting from our cluster analysis are presented in Table 5. The patterns in Table 5 are alternatively depicted in Figure 3, which shows cluster by cluster the implementation levels of each IT security practice measured in this study.

Before analyzing cluster differences, it is worth noting the grand mean of HIT security practices in sampled hospitals. As our security variables are measured through a dichotomous scale (1 if a practice is implemented and 0 if not implemented), the grand mean corresponds to the rate of hospitals that have a given practice implemented. This rate is presented in brackets in the column “variable” of Table 5. Overall, the most implemented practice is the one intended to ensure the confidentiality of electronically-transmitted data (present in 59% of hospitals), closely followed by the practice aiming at guaranteeing the availability of health data in case of a disaster (57%). The less implemented security practice is the access control or the IT workstations that contain sensitive health information (18%). This means that many hospitals tend to overlook the insider threat, which is preoccupying considering that insider threats represent over 50% of IT security breaches and are potentially more devastating than external threats [52,53].

**Table 4:** Regression analyses (dependent variable: information technology security level).

Model	R	R^2^	Adjusted R^2^	Delta R^2^	*F* *(df)*	*P* value
1^a^	.294	.086	.078	.086	10.052 (16)	<.001
2^b^	.346	.120	.111	.034	65.116 (1)	<.001
3^c^	.346	.120	.111	.000	0.104 (1)	.75
4^d^	.348	.121	.111	.001	1.911 (1)	.17

^a^Model 1 predictors: contextual variables (see Table 1).

^b^Model 2 predictors: contextual variables+ transition level toward electronic system.

^c^Model 3 predictors: contextual variables+(transition level toward electronic system)^2^.

^d^Model 4 predictors: contextual variables+(transition level toward electronic system)^3^.

**Table 5:** Health information technology security patterns from cluster analysis. Within rows, different superscripts (*, †, and ‡) indicate significant (*P*<.05) pair-wise differences between means on Tamhane’s T2 (post hoc) test.

Variable (grand mean)		Cluster			Analysis of variance	
		1, n (%)=513 (29.77)	2, n (%)=533 (30.93)	3, n (%)=677 (39.29)	*F* test (*df*)	*P* value
**Confidentiality**						
	Stored data (0.37)	0.53^*^	0.47^*^	0.18^†^	106.6 (2)	<.001
	Transmitted data (0.59)	0.93^†^	1.00^*^	0.00^‡^	9614.0 (2)	<.001
	Access control (0.18)	0.48^*^	0.00^‡^	0.10^†^	303.9 (2)	<.001
Integrity (0.31)		0.78^*^	0.00^‡^	0.20^†^	763.7 (2)	<.001
Availability (0.57)		0.58	0.59	0.54	1.7 (2)	.18

On the basis of Tamhane’s post hoc test (Table 5), we can immediately see that the “availability” criterion does not allow for the discrimination among the 3 clusters: hospitals that have implemented security measures allowing them to immediately recover their EHRs after a disaster are found in almost the same proportions in the 3 clusters (54% to 59%). However, there are differences among the 3 clusters with regard to confidentiality and integrity-related practices.

Overall, the strongest implementers of IT security practices are found in cluster 1, which accounts for 29.77% (513/1723) of surveyed hospitals. The implementation rate of each of the 4 distinctive HIT-related security practices is higher in this cluster than the average rate for all hospitals in our sample.

The weakest implementers of IT security practices are grouped in cluster 3, which comprises 39.29% (677/1723) of the sample. Implementation rates for all IT security practices are lower in this cluster than the overall average rates of the sample. Of note is that none of the 677 hospitals in this group uses encryption to protect electronically-transmitted health records, and only 10.0% of them enforce an access control to HIT systems.

In the middle position comes cluster 2 (533/1723; 30.93%). Although hospitals in cluster 2 have implemented IT security measures related to stored data and transmitted data in higher proportions, none of them have implemented either access control measures or integrity-related practices.

## Discussion

### Health Information Technology Security Index Across Clusters

**Figure 4:**
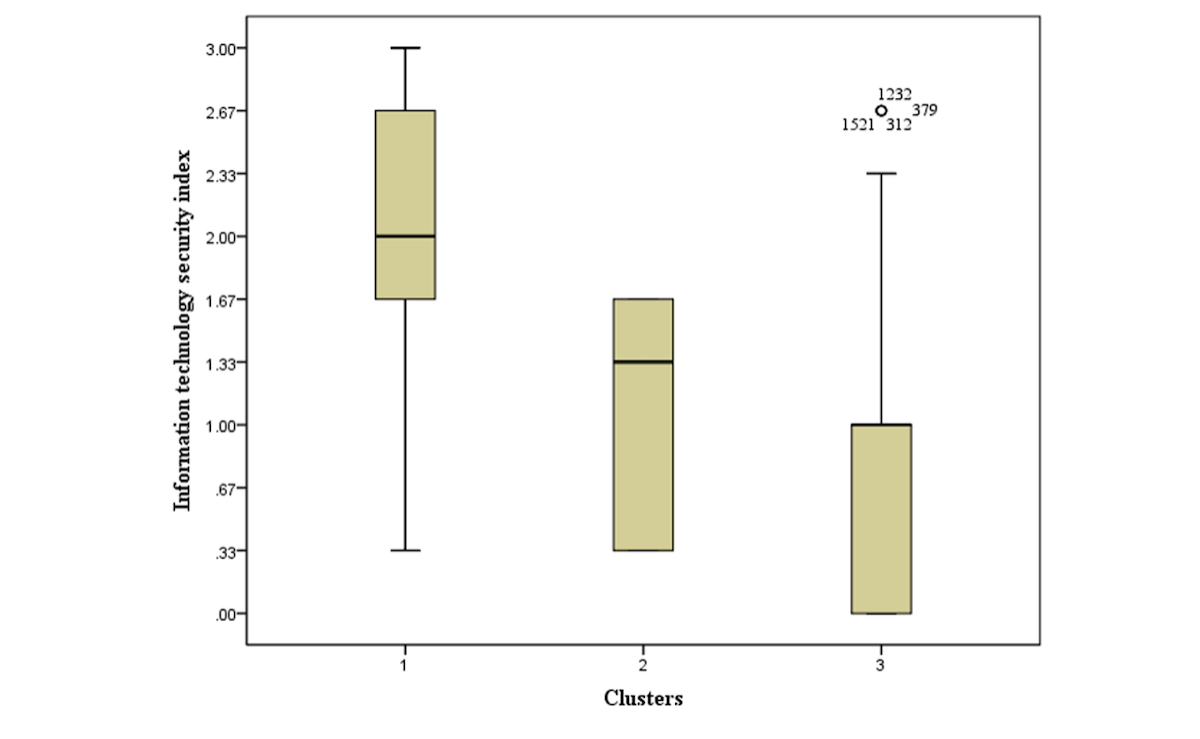
Health information technology security index across clusters. IT: information technology.

The IT security index for all the hospitals in our sample (mean of 1.26) depicts a low level of the implementation of HIT security practices, considering HIT users’ concerns over IT-related security and privacy [7]. The IT security index level varies across the 3 clusters. It is relatively strong (2.00) for cluster 1, very low (0.83) for cluster 3, and low (1.08) for cluster 2. The box plot presented in Figure 4 shows the distribution of observations within each cluster according to the level of the IT security index. Moreover, 50% of hospitals in the strongest cluster (1) display an IT security index equal to or greater than 2, whereas for 25% of them, the IT security index is equal to or above 2.67. Cluster 3 is the only cluster that comprises hospitals with an IT security index that equals zero (25% of hospitals in this cluster). Furthermore, 75% of hospitals in cluster 3 present an IT security index that is equal to or less than 1. The IT security index for hospitals in cluster 2 varies from a minimum of 0.33 to a maximum of 1.67, with 50% of hospitals in this cluster displaying an IT security index between 1.33 and 1.67.

The results from the boxplot are meaningful in that they support our cluster qualification about each cluster’s relative strength or weakness of HIT security implementation. It is particularly interesting to note that despite the gradation that establishes a hierarchy among clusters, allowing a stronger cluster to be identified compared with others in terms of IT security practices implemented, the overall picture does not look very good. The “strong” position of hospitals in cluster 1 is relative. In other words, the strongest hospitals in terms of IT security practices implementation appear so simply because others are badly failing.

### Influence of Context Variables on Health Information Technology Security Patterns

To understand what determines HIT security patterns, we analyzed the contextual variables cluster by cluster. The aim here was to account for the influence of variables “theoretically related to the clusters, but not used in defining clusters” [49].

Multimedia Appendix 2 presents the results of the test of independence (chi-square test of goodness of fit) carried out to assess whether characteristics of hospitals significantly vary according to cluster membership. We do so by comparing the observed and expected distributions of hospitals in different clusters, and the chi-square tests indicate whether or not the observed distributions significantly depart from the expected distribution.

From Multimedia Appendix 2, one can note that only 2 out of 6 hospital characteristics (namely the university affiliation and the size of hospitals) do not allow in any way to significantly discriminate among hospitals’ membership in 1 cluster or another.

The remaining categories of contextual variables present at least 1 characteristic that is significantly overrepresented in 1 or 2 clusters and underrepresented elsewhere. Hospitals in the strongest cluster with regard to HIT security practices (cluster 1) are less likely to fall in the category of “independent/one site” hospitals, and they are underrepresented among hospitals that devote less than 1% of their total budget to the IT function. Conversely, hospitals in cluster 1 are more likely to fall into the category of “independent/multiple sites,” and they are more likely to rely on both national and regional security regulations. On the other end of the security implementation spectrum, hospitals in the weakest cluster (cluster 3) are overrepresented in the category of “independent/one site” hospitals, as well as among hospitals that devote less than 1% of their total budget to the IT function. They are less likely to base their IT security and privacy practices on security regulations at any level (national, regional, and hospital).

The results in relation to the context factors provide important insights and raise some questions. The nonsignificance of the university affiliation status of hospitals goes against the expected results. Previous studies have shown that hospitals' university affiliation is not neutral in their relationship to IT adoption [54] and health data breach risks [55]. This last study shows that health data breaches were more likely to happen in university-affiliated hospitals than in nonaffiliated hospitals. However, our results clearly indicate that the hospitals in our sample are spread across the different clusters (corresponding to different levels of IT security practices) regardless of their university affiliation status. Being at increased risk for health data breaches [55], university-affiliated hospitals should implement IT security measures that are commensurate with their risks.

The nonsignificance of the size of hospitals (number of beds) was also surprising, as one would expect large hospitals to be more aware of HIT security than smaller hospitals: we expected large hospitals to be significantly more represented in cluster 1, which is the strongest with regard to the implementation of HIT security practices. In addition, compared with small and medium-sized hospitals, large hospitals are associated with an increased risk of health data breaches [55], which is one more reason to strengthen their IT security practices.

**Table 6:** Transition toward an electronic-based system and health information technology security patterns.

Transition level	Number of hospitals								
	Cluster 1, n (%)=513 (29.77)			Cluster 2, n (%)=533 (30.93)			Cluster 3, n (%)=677 (39.29)			Chi-square (*df*)	*P* value
	O^a^	E^b^	R^c^	O	E	R	O	E	R		
Paper-dominant, n (%)=214 (12.42)	45	63.7	−18.7	60	66.2	−6.2	109	84.1	24.9	13.4	.001
Hybrid, n (%)=1056 (61.28)	302	314.4	−12.4	329	326.7	2.3	425	414.9	10.1	0.8	.69
Electronic-dominant, n (%)=453 (26.29)	166	134.9	31.1	144	140.1	3.9	143	178	−35.0	14.2	.001

^a^O: observed.

^b^E: expected.

^c^R: residual.

The remaining contextual factors depict a more or less expected picture. The independence status (as opposed to being part of a group of hospitals/care institutions) and the number of sites on which a hospital operates seem to be good predictors of belonging to a weak or strong cluster in terms of implemented IT security practices. Hospitals operating on multiple sites or belonging to a group of other health care institutions need to apply tight IT security measures as their level of exposure is increased, and our results show that there is a trend in that direction. These factors (as well as the university affiliation and the hospital’s size) are structural in nature, and health authorities cannot always act on them to affect IT security practices. However, by understanding their influence on the implementation of IT security practices, health authorities could consider more targeted measures.

Health authorities can act on the level of the hospitals’ IT budget (as a percentage of total hospital budget). Our results suggest that a very low IT budget (in our case, an IT budget less than 1%) is not good for IT security practices implementation. Interestingly, they also suggest that a higher IT budget (more than 3.1%) does not make any difference. The optimal level of IT budget seems to be a level between 1% and 3%. This result raises more questions than answers. First, can we infer from this result that the relationship between the levels of IT budget and the levels of IT security practices implemented is in the shape of an inverted u-curve, meaning that very lower levels of IT budget are as counterproductive as very higher levels? Second, what factors would explain why the range between 1% and 3% is related to stronger IT practices implementation? This question is more challenging given that we do not know the portion of the overall IT budget hospitals in our sample specifically devote to IT security. These questions deserve more attention from researchers, and the answers would provide hospitals with guidance to avoid both under or overinvestment in IT security.

The security regulation constitutes another contextual factor that health authorities can use as a leverage to encourage the implementation of IT security practices in hospitals. It is interesting to note that the adoption of any of the 3 types of security regulations (national, regional, and hospital level) appears to be better (in terms of IT security practices implemented) than nonadoption. However, national and regional regulations seem better than hospital-based security regulations.

### Transition Toward an Electronic-Based System and Health Information Technology Security Patterns

Table 6 presents how hospitals at different phases in their transition toward a fully electronic-based system are distributed across the 3 clusters depicting HIT security patterns. It appears that hospitals in the earlier phases of the transition (paper-dominant hospitals) are less likely to belong to cluster 1 and more likely to belong to cluster 3. Conversely, hospitals that are well-advanced toward electronic-based systems (electronic-dominant hospitals) are more likely to belong to cluster 1 and less likely to belong to cluster 3. On the surface, this seems good news as digitized hospitals are more likely to be found in the strongest cluster with regards to HIT security and privacy practices. However, a closer look at the distribution of hospitals that have an electronic-dominant system across the 3 clusters leads to a less optimistic conclusion: actually, only 36.6% (166 out of 453) of these hospitals belong to cluster 1. This means that there are many hospitals that consider themselves to be well-advanced in their digitization process and that at the same time display major weaknesses in their IT security and privacy practices.

These results are a cause of concern when one considers (1) that the top-ranking risk associated with EHR is the “privacy of data - access control [56]” and (2) that some patients are reluctant to disclose their health information to protect themselves against the perceived EHR privacy and security risks [57].

Ultimately, the consequences of the relative weaknesses in IT security and privacy practices will obscure the benefits expected from the European hospitals’ transition toward fully electronic-based systems.

### Implications and Conclusion

This study highlights a disturbing state in European hospitals regarding the level of implementation of HIT security and privacy practices. Overall, none of the 5 basic security practices investigated in this study is present in more than 60% of surveyed hospitals. Moreover, 3 out of 5 practices are absent in more than two-thirds of the hospitals that were surveyed in this study. These statistics are unsettling as security practices studied here are basic practices that should be implemented in almost all hospitals. Encryption for stored data is used in only 37% of hospitals. It is used for transmitted data in only 59% of hospitals. Many hospitals (more than 80%) do not deem it necessary to control the access to workstations containing health data with health professionals’ cards or codes. There is as few as 18% of hospitals that have implemented these practices. Hospitals in which all these measures are not implemented expose health information to a breach of confidentiality.

In this study, the practices related to integrity and availability are respectively measured at 31% and 57% implementation rates in hospitals. These implementation rates are low for systems containing highly sensitive information. They mean that (1) in almost 70% hospitals, health data in IT systems can be modified by nonauthorized persons, provided they have access to the systems, and (2) more than 40% of surveyed European hospitals would not be able to restore critical clinical information in the aftermath of an incident, resulting in a partial or complete loss of data.

There is another way of looking at our results. Our cluster analysis allowed us to identify 3 patterns of HIT security practices. The majority of surveyed hospitals falls into the 2 worst clusters (clusters 2 and 3): these 2 clusters total 1210 hospitals out of 1723 (70.22%). This means that 7 out of 10 European hospitals are performing poorly in ensuring the security of their EHR.

We expected that hospitals that are well-advanced in their transition toward a fully electronic health system would display higher levels of implementation of IT security practices. Confronting each hospital’s security index (a compounded measure of implemented IT security and privacy practices) to its level of transition toward a fully electronic health system, we unraveled a rather mixed situation, showing that there are many electronically advanced hospitals that are poor implementers of IT security practices. This is great concern not only for hospitals that are poorly equipped in IT security but also for other hospitals with which they share health data and above all for patients whose health information privacy is not adequately protected.

The results presented in this study have theoretical and practical implications. From a theoretical standpoint, it would be helpful to further investigate why the health care sector continues to lag behind in terms of IT security practices implementation. How can one explain the contradiction between the stated importance of IT security measures in hospital settings and the weaknesses in this regard? Are there any sector-related factors that explain the poor implementation of IT security practices in health care organizations? Another research avenue stems from 1 of the limitations of this study. As already mentioned in the Methods section, in the absence of sound evidence suggesting unequal importance of the 3 components of the IT security triad (confidentiality, integrity, and availability), we have assumed an equal weight for each of them. Future research could challenge this assumption and provide empirical-based evidence of the relative importance of the 3 components of the IT security measure. Future research could also determine whether and to what extent this relative importance depends on organizational context. Our results about some context variables, namely the university affiliation status and the size of hospitals, raised an interesting question: why hospitals that present a higher risk profile (university-affiliated hospitals and large hospitals), according to previous research [55], do not appear to be among the hospitals that take relatively strong IT security measures? This question represents an interesting research avenue. Another interesting research avenue is about the determination of the optimal level of IT security investment in hospital settings.

From a practical standpoint, this study raises a red flag that hospitals managers and health policy makers should not ignore. The replacement of paper-based systems with electronic-based systems comes with increased IT-related security risks and requires adequate IT security measures. Thus, hospital managers should make sure to include IT-security practices in their plans toward fully electronic-based systems. For health policy makers, when designing incentives for meaningful use of IT in health care organizations, they should include stringent IT security requirements. Both hospital managers and health policy makers should monitor the digitization process of hospitals to ensure that the implementation of IT security practices keeps pace with the increasing usage of IT.

Although we had access to an interesting dataset collected through a survey by the European Union, we were limited to the questions asked in the survey. This is the problem of using secondary data. We also acknowledge some limits stemming from our definition of security practices. One could enlarge this definition or completely choose other security practices. Besides, we made the assumption of equal weight for all the 3 components (confidentiality, integrity, and availability) of the IT security measure. This is a limitation the reader might take into account when interpreting our results. For the transition level toward a fully electronic-based system, we relied on a self-reported level given by each hospital’s IT manager in the absence of a more objective measure. This can be somehow biased. Despite the abovementioned limits, this paper contributes to the understanding of IT security practices in health care organizations. It also contributes to raising awareness of the security issues that can impede the effective delivery of health care services.

## Appendix

Multimedia Appendix 1 Correlations matrix.

Multimedia Appendix 2 Influence of context variables on health information technology security patterns.

## Abbreviations

C_max_: maximum chance criteria
C_Pro_: proportional chance criteria
eHealth: electronic health
EHR: electronic health record
HIE: health information exchange
HIT: health information technology
ISMG: Information Security Media Group
IT: information technology
