# Supplementary material for: European Hospitals’ Transition Toward Fully Electronic-Based Systems: Do Information Technology Security and Privacy Practices Follow?
Source: JMIR Med Inform. 2019 Mar 25;7(1):e11211. doi: 10.2196/11211 (PMC6452275; doi:10.2196/11211)
Supplement: Multimedia Appendix 1 [file medinform_v7i1e11211_app1.pdf]

	1	2	3	4	5	6	7	8	9	10	11	12	Tol	VIFs
1. Status	1												.90	1.11
2. University Hospital	.14	1											.91	1.11
3. Single/Multiple sites	.05	-.07	1										.95	1.05
4. Size (nb of beds)	-.22	-.26	.027	1									.86	1.16
5. IT budget	.18	.04	.092	-.06	1								.93	1.08
6. National level Security regulation	-.05	-.13	.103	.09	-.04	1							.81	1.23
7. Regional level Security regulation	-.03	-.09	.156	.03	.05	.30	1						.78	1.28
8. Hospital level Security regulation	.04	.02	-.032	.06	.05	-.15	.21	1					.87	1.16
9. Encryption of stored data	.03	-.04	.019	-.10	.06	.09	.15	.06	1				.82	1.23
10. Encryption of transmitted data	.04	-.02	.039	.06	.06	.14	.15	.13	.36	1			.80	1.25
11. Access control	-.03	-.11	.060	.00	.03	.09	.08	.00	.10	.08	1		.94	1.07
12. Integrity	-.06	-.03	-.00	.06	.02	.18	.16	.04	.15	.20	.21	1	.88	1.14
13. Availability	.069	-.02	.03	.04	.13	.05	.08	.06	.12	.09	.04	.05	.96	1.04
