# Supplementary material for: European Hospitals’ Transition Toward Fully Electronic-Based Systems: Do Information Technology Security and Privacy Practices Follow?
Source: JMIR Med Inform. 2019 Mar 25;7(1):e11211. doi: 10.2196/11211 (PMC6452275; doi:10.2196/11211)
Supplement: Multimedia Appendix 2 [file medinform_v7i1e11211_app2.pdf]

Context Variable	Characteristic	Cluster 1 (513; 29.77%)			Cluster 2 (533; 30.93%)			Cluster 3 (677; 39.29%)			Chi-2	P-Value
		O	E	R	O	E	R	O	E	R		
Status	Public	365	356.1	8.9	345	365.3	-20.3	478	466.6	11.4	1.629 (2)	.44
	Private	92	100.4	-8.4	108	103.0	5.0	135	131.6	3.4	1.033 (2)	.60
	Not for Profit	49	49.5	-.5	66	50.7	15.3	50	64.8	-14.8	8.002 (2)	. 02
University Hospital	Yes	83	70.9	12.1	67	73.6	-6.6	88	93.5	-5.5	2.980 (2)	.23
	No	430	442.1	-12.1	466	459.4	6.6	589	583.5	5.5	0.478 (2)	.79
Single/ Multiple sites	Independent/One site	187	211.4	-24.4	210	219.6	-9.6	313	279.0	34.0	7.379 (2)	.03
	Independent/Multiple sites	188	161.4	26.6	168	167.7	.3	186	213.0	-27.0	7.808 (2)	.02
	Part of a group of hospitals	101	101.5	-.5	123	105.5	17.5	117	134.0	-17.0	5.062 (2)	.08
	Part of a group of care institutions	29	23.2	5.8	13	24.1	-11.1	36	30.6	5.4	7.506 (2)	.02
	Other	8	15.5	-7.5	19	16.1	2.9	25	20.4	4.6	5.189 (2)	.08
Size (Number of beds¨)	Fewer than 101 beds	98	112.2	-14.2	119	118.3	.7	161	147.5	13.5	3.037 (2)	.22
	Between 101 and 250 beds	154	152.0	2.0	161	160.2	.8	197	199.8	-2.8	0.070 (2)	.97
	Between 251 and 750 beds	174	173.3	.7	190	182.8	7.2	220	227.9	-7.9	0.560 (2)	.76
	More than 750 beds	70	58.5	11.5	53	61.7	-8.7	74	76.9	-2.9	3.599 (2)	.17
IT Budget (% of Total Hospital Budget)	Less than 1%	131	152.0	-21.0	150	155.4	-5.4	205	178.6	26.4	6.991 (2)	.03
	Between 1% and 3%	211	187.6	23.4	194	191.9	2.1	195	220.5	-25.5	5.891 (2)	.05
	Between 3.1% and 5%	37	38.1	-1.1	43	39.0	4.0	42	44.8	-2.8	0.616 (2)	.74
	More than 5%	15	16.3	-1.3	16	16.6	-.6	21	19.1	1.9	0.314 (2)	.86
Security regulation	National level	347	303.0	44.0	311	309.6	1.4	339	384.4	-45.4	11.758 (2)	.003
	Regional level	188	144.4	43.6	154	147.5	6.5	133	183.2	-50.2	27.212 (2)	.000
	Hospital level	367	352.2	14.8	391	359.9	31.1	401	446.9	-45.9	8.024 (2)	.02

O: Observed; E: Expected; R: Residual.
